# Aptamers: Problems, Solutions and Prospects

**Published:** 2013

**Authors:** A.V. Lakhin, V.Z. Tarantul, L.V. Gening

**Affiliations:** Institute of Molecular Genetics, Russian Academy of Sciences, 2 Kurchatov Sq., Moscow, Russia, 123182

**Keywords:** SELEX, aptamer, diagnostics, therapeutics, problems

## Abstract

Aptamers are short single-stranded oligonucleotides that are capable of binding various molecules with high affinity and specificity. When the technology of aptamer selection was developed almost a quarter of a century ago, a suggestion was immediately put forward that it might be a revolutionary start into solving many problems associated with diagnostics and the therapy of diseases. However, multiple attempts to use aptamers in practice, although sometimes successful, have been generally much less efficient than had been expected initially. This review is mostly devoted not to the successful use of aptamers but to the problems impeding the widespread application of aptamers in diagnostics and therapy, as well as to approaches that could considerably expand the range of aptamer application.

## INTRODUCTION

Nucleic acids (NAs) were for a long time regarded only as compounds whose major functions were related to the storage of inherited information (DNA) and its transfer from gene to protein (RN A). However, as time has passed, new functions, such as enzymatic catalysis (performed by ribozymes) and transcription regulation, have been reported. The increasing number of such examples has forced the scientific community to reconsider its original opinion about the functions of NAs and to propose the so-called “RN A world theory” [1, 2]. According to this theory, NAs can perform very diverse functions and have probably ensured all the catalytic reactions for the period since life took hold on our planet [3]. The discovery of oligonucleotides that can specifically bind various target molecules and are known as aptamers was a valuable contribution to confirming the multifunctional nature of NAs [4, 5].

Aptamers are small (usually from 20 to 60 nucleotides) single-stranded RN A or DNA oligonucleotides able to bind target molecules with high affinity and specificity. Currently, a large number of generated aptamers can bind various targets, ranging from simple inorganic molecules to large protein complexes, and entire cells. In fact, aptamers are nucleotide analogues of antibodies, but aptamer-generation is significantly easier and cheaper than the production of antibodies [6, 7]. Moreover, aptamers are neither immunogenic nor toxic [8]. All these features make aptamers ideal candidates for diagnostic and therapeutic applications, purification of target molecules from complex mixtures, biosensor design, etc. [9, 10]. Aptamers are so widely applicable that new aptamer-related reports are published almost every day. A special database has been created (http://aptamer.icmb.utexas.edu) to classify the aptamer-related data and provide access to information about numerous, existing aptamers.

The basic methods used to engineer aptamers were described over 20 years ago [11, 12]. Aptamers are usually selected from the oligonucleotide collection that is known as the initial oligonucleotide pool (IOP) and includes 1014-1015 different oligonucleotides. IOP is often called a “combinatorial library.” This comparison is not quite accurate, since such a library contains all possible oligonucleotides of selected size by definition and is too big for practical purposes (a relatively small library contains about 1018 different oligonucleotides). IOP is an aliquot of the synthetic chemical combinatorial library and contains single-chained DNA or RN A oligonucleotides conditioned for binding to the target molecule. Oligonucleotides composing IOP include 30- to 50-nucleotide-long variable parts (each position can be occupied by one of four nucleotides). Variable parts of aptamers are flanked by constant fragments to make the necessary manipulations (such as amplification and transcription) possible. It should be noted that RN A aptamers provide a significantly greater structural diversity compared to DNA aptamers, but their application is fraught with problems (RN A molecules are easily degradable by different factors, such as RN ases, high temperature, alkaline medium etc.) [13, 14].

**Fig. 1:**
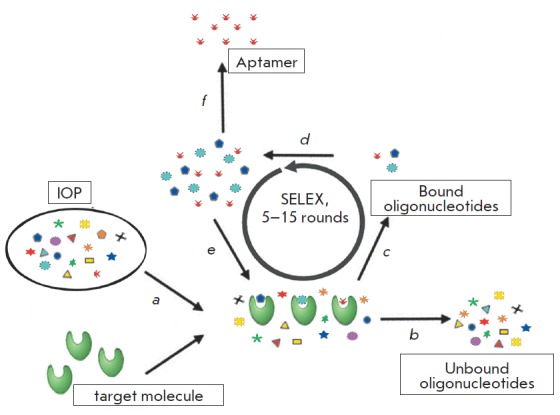
Scheme of SELEX. (a) IOP is incubated with a target molecule. (b) Unbound oligonucleotides are separated from bound molecules by washing steps. (c) Bound oligonucleotides are eluted from the target molecule. (d) Eluted oligonucleotides are amplified using the PCR (DNA-SELEX) or RT-PCR (RNA-SELEX) technique. (e) The enriched pool is then subjected to further rounds of selection. (f) After 5–15 rounds, aptamers are cloned and analyzed in detail

The conventional method for aptamer engineering known as SELEX (systematic evolution of ligands by exponential enrichment) can be conditionally separated into two alternating stages (*Fig. 1*). At the first stage, the original oligonucleotides are amplified by a polymerase chain reaction (PCR ) to the desired concentration. In case of selection of RN A aptamers, the pool of single-chained oligoribonucleotides is generated by *in vitro *transcription of double-stranded DNA with T7 RN A-polymerase. For the selection of DNA aptamers, a pool of single-stranded oligodeoxyribonucleotides is generated by strand separation of double-stranded PCR products. At the second stage, the amplified pool is incubated with target molecules and interacting oligonucleotides are used for the first stage of the next SELEX round [7, 15].

Separation of oligonucleotides with higher affinity for target molecules and removal of unbound oligonucleotides are achieved through intense competition for binding sites. The selection pressure rises with every SELEX round. Maximum enrichment of the oligonucleotide pool with aptamers with the strongest affinity for the target molecule is usually achieved after 5-15 rounds [16, 17]. The SELEX method is applicable not only to the selection of aptamers capable of binding target molecules, but also to the selection of oligonucleotides with a particular enzymatic activity. In this case, the ability to catalyze the desired chemical reaction is used as a selection criterion [18, 19].

## LIMITATIONS IN APTAMER APPLICATION AND POSSIBLE SOL UTIONS

The use of aptamers is fraught with problems that will be discussed in this review. The main bottlenecks limiting the wide application of aptamers are described below.

**Problem 1. Aptamer degradation**

The rapid degradation of aptamers (especially RN A aptamers) by nucleases in biological media, and in blood in particular, is a serious problem that puts limits on their practical application. The average time of oligonucleotide decay in blood ranges from several minutes to several tens of minutes depending on the oligonucleotide concentration and conformational structure. Since such a short time range is unacceptable for most therapeutic applications, several methods for protecting aptamers against degradation by nucleases have been developed.

**Fig. 2:**
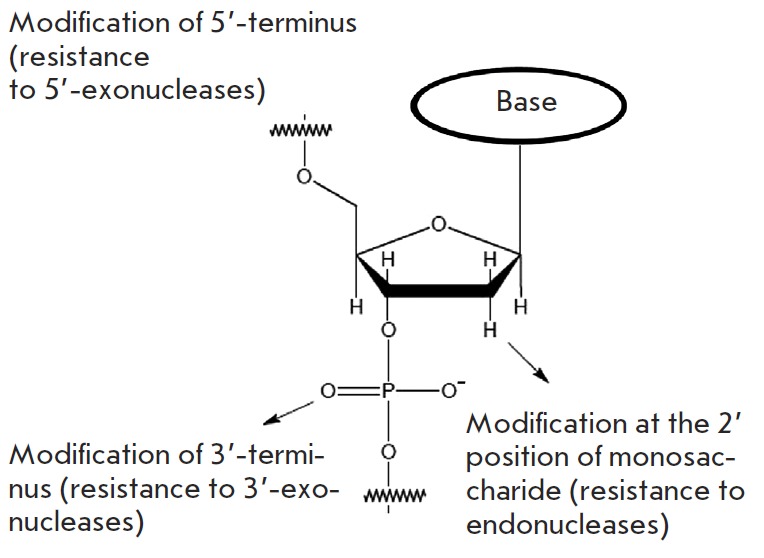
Most frequently used modifications of nucleotides providing resistance of aptamers to nuclease degradation

**Fig. 3:**
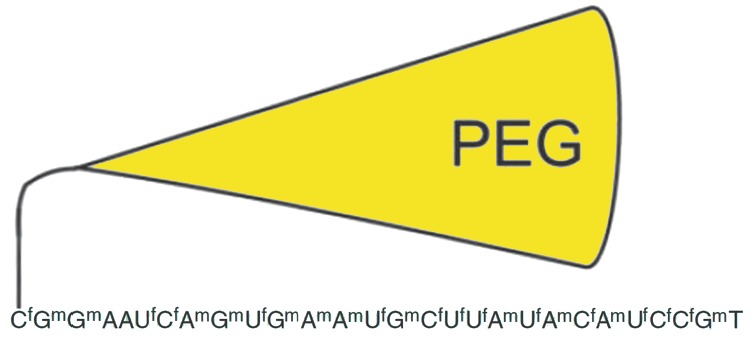
The structure of the first FDA-approved aptamer, Macugen. The following modified nucleotides were used: f – 2'-fluoronucleotide, m – 2'-O-methylnucleotide. The aptamer was conjugated to 40 kDa PEG to avoid quick excretion during renal filtration

One of the conventional methods used to generate nuclease-resistant aptamers is by performing SELEX with oligonucleotides containing modified nucleotides (*Fig. 2*). Special DNA and RN A polymerases that are able to utilise nucleoside triphosphate substrates with a modified, for example, 2’ sugar position are used to generate such oligonucleotides. 2’-Amino pyrimidine nucleosides [20, 21], 2’-fluoropyrimidine nucleosides [22, 23], 2’-O-methyl purine, and 2’-O-methyl pyrimidine nucleosides [24, 25] are currently used for this purpose. The only aptamer approved for medical application known as Macugen (Fig. 3) is a vivid example of an oligonucleotide modified using this approach [26]. Modification of nucleotides already included into aptamers could also be performed after the SELEX procedure; however, the inclusion of additional functional groups in this case can affect the specificity and affinity of an aptamer. Nevertheless, some modifications can increase aptamer resistance to nucleases without affecting their binding to target molecules. The most common and effective type of such aptamer improvements is the modification of 3’- and 5’-nucleotides [27]. Sometimes unmodified aptamers demonstrate very high resistance to degradation by blood nucleases [28]. This feature might be provided by the formation of specific three-dimensional structures that protect the 3’- and 5’-termini of aptamers against exonucleases.

The closed ring structures emerging after ligation of the 3’- and 5’-termini of the same aptamer are also highly resistant to degradation by nucleases. Several different aptamers can also be ligated to a closed structure with multiple specificities [29, 30]. The generation of such ring structures is an optimal approach for the regular injection of high amounts of aptamers, since the degradation products of some modified oligonucleotides have the potential of being toxic [31].

The novel approach to avoiding aptamer degradation by nucleases was provided by the development of “mirror aptamers” (Spiegelmers), which have an oligonucleotide backbone composed entirely of *L*-ribose (RN A spiegelmers) or *L*-deoxyribose (DNA spiegelmers). The development of spiegelmers was favored by the fact that nucleases effectively cleave only *D*-, but not the unnatural *L*-oligonucleotides. However, if an aptamer with a known target is re-synthesized from* L*-nucleotides, this new aptamer will bind only an unnatural enantiomer protein containing *D*-amino acids. This problem can be solved if the primary selection of aptamers composed of *D*-nucleotides is performed using a synthetic *D*-protein. When selected aptamers are sequenced, they can be re-synthesized as spiegelmers binding a natural *L*-protein. Such spiegelmers are very stable and almost fully resistant to enzymatic degradation [32, 33].

**Fig. 4:**
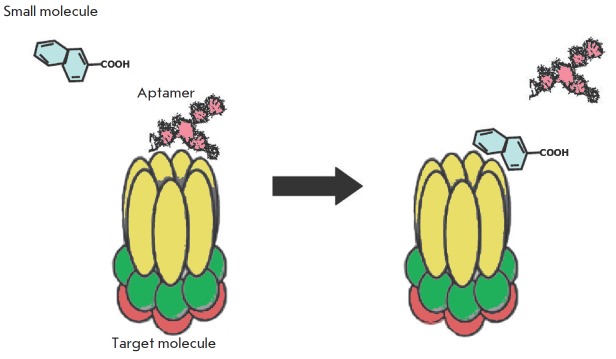
Aptamer displacement screening. This approach allows one to select small molecules competing with an aptamer for the same binding site

Another approach to avoiding the problems related to aptamer degradation is by the recently developed method known as “aptamer displacement screening.” This method is based on the screening of low-molecular- weight substances according to their ability to displace aptamers from the binding site of a target molecule (Fig. 4). It is presumed that the selected substance will have specificity and affinity similar to those of the displaced aptamer. The inhibitory effect of these lowmolecular- weight compounds on protein targets is often identical to the effect of aptamers [34, 35].

**Problem 2. Aptamer excretion from the bloodstream by renal filtration**

The removal of aptamers from the bloodstream via renal filtration complicates their therapeutic application. Most aptamers have a molecular weight ranging from 5 to 15 kDa (15-50 nucleotides), and they can be easily excreted by kidneys capable of removing substances with a molecular weight below 30-50 kDa. Conjugation of aptamers with polyethylene glycol (PEG) with a molecular weight of 20 or 40 kDa is the most common solution to this problem (Fig. 3). This method is currently being used to increase the bloodstream circulation time not only of oligonucleotides, but also of proteins, peptides and low-molecular-weight substances [36, 37]. The PEG-conjugated aptamers are excreted from the bloodstream slowly (up to several days) and do not lose their specificity. And, besides, such PEG-conjugated aptamers are more effectively delivered to tissues and organs [38, 39]. As an alternative, aptamers could also be conjugated with cholesterol molecules. This modification also prolongs aptamer circulation in the bloodstream [40].

**Problem 3. Control of the duration of action**

**Fig. 5:**
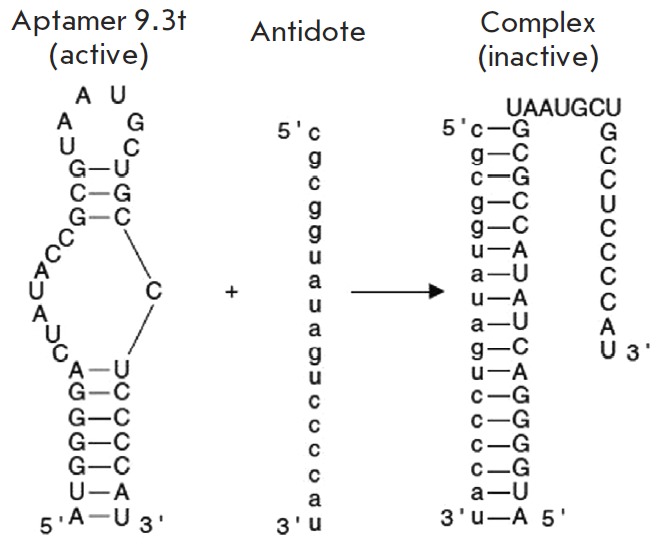
Antidote-dependent regulation of aptamer functioning. The aptamer 9.3t is shown as an example [77]. This aptamer interacts with the coagulation factor IXa and has anticoagulation properties. Administration of a complementary antidote leads to quick inactivation of this aptamer and restoration of blood coagulation

The pharmacokinetic parameters of a drug (e.g., action duration) are very important in its therapeutic application. The duration of action depends on multiple factors, including degradation, involvement in metabolic processes, renal excretion, etc. All these factors should be taken into consideration before drug prescription, and sometimes they limit its application. The use of aptamers as drugs can often solve the problems associated with controlling the duration of action. One of the possibilities is to generate antidotes to aptamers by synthesizing a complementary oligonucleotide. Hybridization with antidote causes a change in aptamer conformation and complete loss of its ability to bind the target molecule (Fig. 5). The efficiency of this approach has been confirmed by experiments on animal models. An aptamer was delivered into the bloodstream and exhibited a therapeutic effect, while subsequent injection of an antidote inactivated the aptamer and stopped its action [41, 42]. The high efficiency of aptamer hybridization with an antidote in blood provides a unique opportunity to control the duration of the therapeutic action. It makes the application of aptamers preferable, since it is either impossible or very difficult to control the duration of action of antibodies or low-molecular-weight substances.

Another effective and inexpensive way to control aptamer activity in the bloodstream without the necessity to generate a unique antidote is through the application of polycationic biopolymers that effectively bind polyanionic oligonucleotide molecules [43, 44]. Numerous polymers originally developed for gene therapy and delivery of DNA or siRN A possess the ability to bind NAs [45, 46]. Some low-molecular-weight molecules, such as porphyrin, can also bind particular conformational structures and inactivate an aptamer [47]. The blood does not contain significant amounts of NA due to the high activity of nucleases; therefore, it is presumed that biopolymers will bind preferentially foreign NAs (in particular, aptamers).

Another approach to controlling aptamer activity is inducible activation, i.e. conversion of an aptamer in an inactive form to an active one. For example, an inactive aptamer containing nucleotides with particular photosensitive modifications does not bind the target molecule. After being exposed to light with a particular wavelength, the aptamer loses its photosensitive groups and is converted into a functionally active state. This approach allows one to control both the time and site of aptamer activation [48, 49].

**Problem 4. Interaction of aptamers with intracellular targets**

Most aptamers were selected using molecules located on the cell surface or in the bloodstream. This potentially makes their application rather easy, since all that is needed to trigger the therapeutic effect is to deliver the aptamer into the bloodstream. However, some advances in the intracellular delivery of aptamers have recently been achieved. Special expression systems are able to generate aptamers inside cells and ensure their accumulation either in nucleus or in the cytoplasm. For example, transfection of cells with a recombinant vector expressing the aptamer sequence under a U6 promoter allows specific inactivation of nuclear target proteins [50, 51], while aptamer expression under a tRN A promoter ensures predominantly cytoplasmic localization of aptamers [52]. Cell-type-specific aptamer synthesis can be achieved by using directional viral expression systems that deliver vectors to particular cells [53, 54]. The concentration of expressed aptamers (also known as intramers) can be increased not only by using strong promoters that ensure a high level of expression, but also by limiting the rate of aptamer degradation by nucleases through protection of the 3’- and 5’- termini with additional structures (e.g., hairpins) [50].

Another way of delivering aptamers to intracellular target molecules is by the transfer of aptamers from the bloodstream to cells through receptor-dependent endocytosis [55, 56]. For example, endocytosis of aptamer binding prostate-specific membrane antigen (PSMA) provides effective and specific delivery of conjugated drugs to cancer cells expressing this antigen on their surface [57, 58].

**Problem 5. Generation of aptamers using unpurified target proteins**

Aptamer generation in most cases requires the availability of purified target molecules. Protein target molecules are expressed in cell cultures and purified by affine chromatography. These procedures are timeand labor-consuming, thus delaying the production of corresponding aptamers. Moreover, some proteins are difficult to purify due to their chemical properties. Sometimes aptamers generated against target proteins expressed in prokaryotic cells do not interact with the same proteins expressed in eukaryotic cells due to post-translational modifications. These modifications can make epitopes of eukaryotic proteins inaccessible to aptamers generated against the proteins expressed in prokaryotic cells [59].

**Fig. 6:**
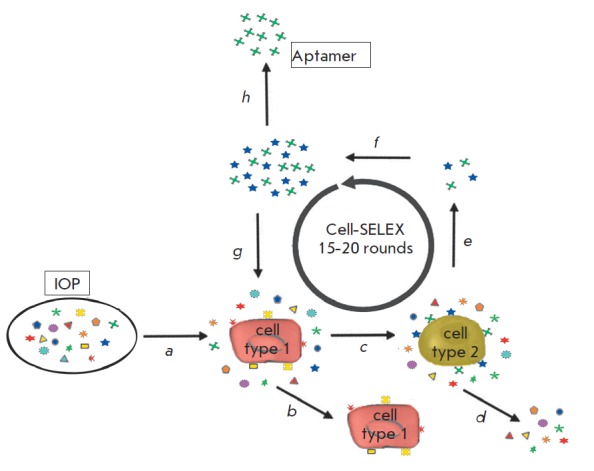
Scheme of Cell-SELEX. (a) IOP is first incubated with a nontarget cell in a negative selection step. (b) All oligonucleotides that show binding to the negative control cells are removed. (c) Unbound oligonucleotides from the negative step are added to the target cells in a positive selection step. (d) Unbound oligonucleotides from the positive step are separated from bound molecules by washing steps. (e) Oligonucleotides binding target cells are subsequently eluted. (f) Eluted oligonucleotides are amplified using the PCR (DNA-SELEX) or RT-PCR (RNA-SELEX) technique. (g) The enriched pool is then subjected to further rounds of selection. (h) After 15–20 rounds, aptamers are cloned and analyzed in detail

The modified SELEX protocol (Cell-SELEX) can be used to select aptamers that recognize cell-surface proteins [60, 61] (Fig. 6). Cell-SELEX allows to select aptamers located directly on the surface of live cells. It is also possible to generate aptamers that recognize specific microorganisms (e.g., such parasites as trypanosomes) [62, 63]. Cell-SELEX includes a negative selection step with a cognate cell type or cell line negative for target markers. One of the advantages of Cell-SELEX is that it does not require exhaustive information about cell-specific protein markers. The combination of negative selection with normal cells and positive selection with transformed cells will provide aptamers specific to tumor markers and promote the development of early cancer diagnostics.

The mutations that cause cancer first change the expression patterns, while the morphology of cells and tissues is changed later. The conventional methods of cancer diagnostics are focused mainly on morphological abnormalities and cannot recognize the early stages of cancer. This problem can be solved with Cell-SELEX- generated aptamers that recognize cancer cells. Aptamer microarrays can find trace amounts of cancer cells in the bloodstream [64, 65]. Marker-specific aptamers conjugated to gold particles are successfully used as contrasting agents for cancer-type specific diagnostics [66, 67].

New methods for the selection of aptamers that recognize intracellular target proteins in cell extracts have been developed [68, 69]. The negative selection step with extract from cognate cells lacking the target protein is included in SELEX when the target concentration is low. The resulting aptamer pool will be enriched in oligonucleotides that recognize the target protein. The negative selection step is unnecessary for target proteins with a high (1-10%) concentration [68, 69]. This SELEX modification allows fast generation of aptamers that recognize cell-type specific intracellular proteins. Target proteins can be further purified in native form by means of affinity chromatography on selected aptamers [70]. This approach can be useful for the analysis of purified enzymes, since fusion with affinity tags (GST, His, etc.) can unpredictably change enzyme properties [71].

Tissue-specific aptamers can be selected using a new approach known as *in vivo *SELEX [72]. A pool of nuclease- resistant aptamers is injected into the bloodstream of an organism containing a specific tissue (e.g., tumor). This tissue is later excised; the aptamers are extracted, amplified, and re-injected into the target organism. Several rounds of such selection generate a pool of aptamers that target *in vivo *specific tissue. Many of these aptamers can migrate into cells and bind intracellular targets [72]. *In vivo *SELEX provides another significant advantage: the generated aptamers do not bind to blood or cell-surface proteins.

**Problem 6. Aptamer cross-reactivity**

Regardless of their high specificity, aptamers that recognize particular targets can also bind to molecules with a similar structure. Four aptamers against DNApolymerase β generated in our laboratory can also bind and inhibit DNA polymerase κ, which belongs to another DNA polymerase family [73]. Aptamer cross-reactivity can be an obstacle to their therapeutic application because of the possible side effects caused by aptamer interaction with other proteins; however, this problem can be avoided by introducing a SELEX negative selection step with structurally similar molecules. The results obtained in our laboratory confirm the efficiency of this approach. A more stringent SELEX protocol was used to produce a highly specific aptamer against DNA polymerase ι. This aptamer can bind neither to DNA polymerases κ nor to β [74].

**Problem 7. Automation of aptamer generation**

Generation of aptamers seems to be a rather simple protocol, but in reality it is a time- and labor-consuming process. The selected aptamers sometimes turn out not to have the best affinity and specificity due to a suboptimal SELEX procedure. Automated SELEX [75, 76] allows one to avoid these problems and to generate aptamers with the required qualities within several days.

Another new method known as CE -SELEX (capillary electrophoresis SELEX) includes a modified stage of selection of target-bound oligonucleotides and allows to generate aptamers in one round. Nonequilibrium capillary electrophoresis of equilibrium mixtures (NECEE M) is used for aptamer fractioning. The entire selection procedure lasts 1-2 days and allows to select aptamers with strictly specified binding parameters *K*_d_,* K*_off_ and *K*_on_ [77, 78].

## CURRENT STATUS OF APTAMERS IN DIAGNOSTICS AND THERAPHY

Mono- and polyclonal antibodies are routinely used for the diagnostics of various diseases. However, they can sometimes be successfully replaced by aptamers, especially when effective and specific binding to a target molecule is required [79, 80]. Aptamers can recognize a membrane-immobilized protein in Western blotting protocols more effectively than antibodies can [81, 82]. ELISA protocols are also more sensitive when aptamers are used instead of antibodies [83, 84]. Similar to antibodies, aptamers can be used to purify target proteins [85, 86]. In contrast to antibodies, aptamers can be selected against non-immunogenic and toxic substances [87, 88].

Aptamers are also used as recognizing elements in biosensors [89, 90]. They are 10-100 times smaller than antibodies and can be arranged with a higher density on the biosensor surface. Aptamer-based biosensors require a smaller volume of the tested sample and can be re-used without loss of sensitivity [91, 92].

Aptamers are promising therapeutic agents, because they are cheap, non-immunogenic, and easy to modify. Inhibition of target enzymes is the main field of aptamer application as drugs. Aptamers inhibit target enzymes by binding to the catalytic center or inducing conformation changes in a protein’s structure [93, 74]. However, when an aptamer is similar to an activating ligand, it can induce enzyme activation [94, 95].

Aptamer-based protocols of treatment of viral diseases are under development. Aptamers that recognize many viruses, including the human immunodeficiency virus (HIV), hepatitis C virus (HCV) and influenza virus, are already available [96, 97]. Aptamers can efficiently bind and inhibit many important viral enzymes, including reverse transcriptases, integrases, etc. However, the problem of effective delivery of aptamers or aptamerexpressing vectors into cells has yet to be solved. Nevertheless, aptamers can effectively bind viral capsid proteins. Such binding inhibits the interaction between viruses and cellular receptors and prevents viral entry into the cell [98, 99]. It makes the potential application of aptamers for antiviral prophylaxis or therapy much easier: aptamers can be injected intravenously or applied on the skin as a solution or ointment.

Aptamers against cell-type specific protein markers can be conjugated to drugs for targeted delivery. The following drug types can be used for conjugation to aptamers:

Toxic and radioactive substances that are inapplicable in therapy at high doses. They can be conjugated with aptamers and injected in low doses. These substances will subsequently concentrate locally (e.g. in tumor) to reach therapeutic doses [100, 101];

Easily degradable or excretable substances (e.g., siRNA). Cell- or tissue-specific delivery of siRN A conjugated to an aptamer removes the major obstacle to the therapeutic siRN A application [102, 103];

Drug-loaded nanoparticles. Animal models demonstrate the low efficiency of targeted delivery of nanoparticles conjugated to anti-tumor antibodies. These bulky conjugates are quickly removed from the bloodstream by phagocytes and demonstrate the low efficiency of delivery into solid tumors. Conjugates of nanoparticles with aptamers are significantly smaller and show better tissue penetration [104, 105]. The use of aptamer-conjugated liposomes for targeted drug delivery into cancer cells is the most promising area in this research; it has already proved efficient in some cases [106, 107]; and

Endogenous enzymes. Intracellular delivery of enzyme- aptamer conjugates can be used to restore the functional activity of cells if these enzymes are absent or dysfunctional [108].

## CONCLUSIONS

Aptamers are a special class of substances that combine the advantages typical both of low-molecular-weight substances and proteins. Aptamers demonstrate an affinity and specificity similar to those of monoclonal antibodies. Meanwhile, aptamers are non-immunogenic and demonstrate high tissue penetration similar to that of small molecules. However, aptamers have not been commonly used thus far. The aptamer generation protocol SELEX was developed over 20 years ago, but only one aptamer, Macugen (or Pegaptanib), has been approved for therapeutic application (Fig. 3). Macugen binds to the vascular endothelial growth factor (VEGF) and blocks abnormal angiogenesis in the eye, thus preventing intraocular hemorrhage and loss of vision [26, 109].

Although aptamers have a number of advantages, it may seem rather strange that their share among modern therapeutic drugs is rather low. Aptamers are recently engineered substances, and this fact explains their rare application as therapeutic agents. For example, monoclonal antibodies were developed in 1975, but it was not until 1986 that the first antibody-based drug was approved by the U.S. Food and Drug Administration. The second drug of the type reached the pharmaceutical market in 1994, and now about twenty antibody- based drugs are used in clinic. The clinical trials may last for over 10 years and cost hundreds of millions of U.S. dollars. On the other hand, the first annual sales of Macugen (in 2005) have already exceeded 200 million U.S. dollars, a good incentive for the development of new aptamer-based drugs.

The use of aptamers in diagnostics has fewer limitations related to health risk, since there is no direct health risk in this case. In our opinion, the main obstacle to aptamer use in diagnostics is related to the lack of standardized protocols. The different aptamers generated in the same laboratory against the same target will differ in their primary structure, affinity, specificity, and other chemical parameters. As a consequence, the protocol developed for one aptamer might appear inapplicable for another oligonucleotide. This circumstance creates a problem for aptamer application in the diagnostic of human diseases, which can be solved by generating standardized kits and protocols based on well-characterized aptamers with optimum characteristics. The constantly falling cost of chemical synthesis and generation of databases of characterized aptamers make this unification possible in the nearest future.

Almost all problems related to aptamer application have been solved to a certain extent, and we hope that these new substances will soon find extensive use both as scientific tools and as diagnostic and therapeutic agents.

## Abbreviations

NAs: nucleic acids
IOP: initial oligonucleotide pool
PEG: polyethylene glycol
SELEX: systematic evolution of ligands by exponential enrichment
siRNA: small interfering RNA
